# Location of out-of-hospital cardiac arrests and automated external defibrillators in relation to schools in an English ambulance service region

**DOI:** 10.1016/j.resplu.2022.100279

**Published:** 2022-07-26

**Authors:** Madeleine Benson, Terry P. Brown, Scott Booth, Felix Achana, Christopher M. Smith, Gill Price, Matt Ward, Claire Hawkes, Gavin D. Perkins

**Affiliations:** a Heartlands Hospital, Birmingham B9 5SS, UK; b Applied Research Collaboration West Midlands, Warwick Clinical Trials Unit, University of Warwick, Coventry CV4 7AL, UK; c Warwick Clinical Trials Unit, University of Warwick, Coventry CV4 7AL, UK; d West Midlands Ambulance Service University NHS Foundation Trust, Brierley Hill DY5 1LX, UK

**Keywords:** Out-of-Hospital Cardiac Arrest, Bystander defibrillation, Automated External Defibrillator, Public access defibrillation, Schools

## Abstract

**Introduction:**

This study sought to identify the availability of automated external defibrillators (AEDs) in schools in the region served by West Midlands Ambulance Service University NHS Trust (WMAS), United Kingdom, and the number of out-of-hospital cardiac arrests (OHCA) that occurred at or near to schools. A secondary aim was to explore the cost effectiveness of school-based defibrillators.

**Methods:**

This observational study used data from the national registry for OHCA (University of Warwick) to identify cases occurring at or near schools between January 2014 and December 2016 in WMAS region (n = 11,399). A school survey (n = 2,453) was carried out in September 2017 to determine the presence of AEDs and their registration status with WMAS. Geographical Information System mapping software identified OHCAs occurring within a 300-metre radius of a school. An economic analysis calculated the cost effectiveness of school-based AEDs.

**Results:**

A total of 39 (0.34%) of all OHCAs occurred in schools, although 4,250 (37.3%) of OHCAs in the region were estimated to have occurred within 300 metres of a school. Of 323 school survey responses, 184 (57%) had an AED present, of which 24 (13.0%) were available 24 h/day. Economic modelling of a school-based AED programme showed additional quality-adjusted life years (QALY) of 0.26 over the lifetime of cardiac arrest survivors compared with no AED programme. The incremental cost-effectiveness ratio (ICER) was £8,916 per QALY gained.

**Conclusion:**

Cardiac arrests in schools are rare. Registering AEDs with local Emergency Medical Services and improving their accessibility within their local community would increase their utility.

## Highlights

• Out-of-hospital cardiac arrest (OHCA) remains low both in England and worldwide.
• About a third of all OHCAs occur within 300 m of a school.
• Considering the usage rates of public access defibrillators there is significant potential for increasing their use.
• Improving access to automated external defibrillators (AED) in schools and their registration with Emergency Medical Services could lead to greater use.
• A strategy for placing AEDs in schools is likely to be cost-effective.

## Introduction

Survival from out-of-hospital cardiac arrest (OHCA) remains low both in England and worldwide1., 2., 3., 4.. In 2014, of the 28,729 OHCAs where resuscitation was attempted by the English Emergency Medical Services (EMS), survival was 7.9%^2^; more recently it is about 9%3., 4.. Even though survival is more likely in those who received bystander cardiopulmonary resuscitation (CPR) and early defibrillation, the use of publicly available automatic external defibrillators (AEDs) in England remains low at between 2%2., 5. and 8%^3^.

Resuscitation Council UK (RCUK) guidance suggests that an AED should be available within 5 minutes of a witnessed arrest.^6^. Assuming a brisk walking speed of 4mph, roughly 100 m/min, a bystander could travel around 500 m in five minutes. Even when the EMS know of a registered AED within walking distance of an OHCA, unless available 24 h/day, at certain times that AED will be inaccessible. This loss of accessibility has been shown to be as high as 39.7% in Canada^7^ and 66.7% in Denmark^8^ in school-based AEDs.

A systematic review suggested that OHCAs at school are rare, accounting for between 0.15–0.25% of OHCAs, and are more likely to happen in adults than in children^9^. More recently, the figure for England is around 0.1% (3, 4), and data collected by the European Registry of Cardiac Arrest project (EuReCa) indicates a similar figure, with less than five patient (out of 23 cases) being aged under 17-years^10^. Data published by the International Liaison Committee on Resuscitation (ILCOR) Research and Registries Working Group indicates it’s about 0.4–0.6%^11^ although this was based on data from only two countries. However, studies have found higher rates of both ventricular fibrillation (VF)12., 13. and survival13., 14. in OHCAs occurring in any age group on school premises. The survivors also have a more favourable neurological prognosis15., 16.. These findings may be due to the supervised environment leading to more witnessed OHCAs and the likelihood of staff being trained in Basic Life Support.

Since 2014, the Department for Education (DfE) in England has recommended that schools consider purchasing an AED^17^. They published guidance on the installation and maintenance of AEDs and made arrangements for nurseries, schools and colleges in England to be able to purchase AEDs at a reduced cost^18^.

The aim of this study was to investigate the geographical relationships between locations of OHCAs, schools and AEDs in the West Midlands. Our objectives were to establish the incidence and demographics of OHCAs that occurred in and around schools in the West Midlands, to identify the number of schools with an AED on site that were known to the local ambulance service, to determine the AED coverage of OHCAs, and calculate the cost-effectiveness of placing AEDs in schools.

## Methods

### Study area

We conducted an epidemiological observational study of the region covered by the West Midlands Ambulance Service University NHS Foundation Trust (WMAS) who serve a population of 5.8 million people, and cover an area of over 13,000 km^2^ (https://wmas.nhs.uk).

### Out-of-hospital cardiac arrest data

OHCA data was retrieved from the OHCA Outcomes (OHCAO) registry at the University of Warwick containing details of all OHCAs where EMS started or continued resuscitation attempts. Details of the OHCAO registry have been previously published^19^.

We analysed the anonymised details of OHCAs occurring in the West Midlands between January 2014 and December 2016, including event location, date and time, age of case, public access defibrillator (PAD) availability/usage and survival. OHCAs that occurred on school premises were identified by analysing information provided in the event location and if the Utstein location was given as ‘Educational Institution’. Educational institution could include Universities, however, we cross-checked with the event location to ensure these were not included in the analysis.

### Schools’ data

Postal addresses of 2634 schools in the West Midlands region were gathered from the Department of Education’s Edubase, the most recent register of state-funded and independent schools in the area^20^. No address details were located for 3 remaining schools, accounting for less than 10 pupils each, which were excluded. The age-range of students at the schools was 6–18 years. Universities were not surveyed.

Schools were contacted via email and asked whether they had an AED on site and, on reply, about availability and registration status of the AED. We took the median reported opening and closing hours of schools who participated in the survey as an estimate for schools across the West Midlands. In September 2017 the first round of emails contacted 2453 (93%) schools. Three rounds of emails were sent. Schools were able to opt out of further contact.

### Public-access AED locations

We were granted access to the database of PADs registered with WMAS on 23/10/17. The database does not contain details of every AED in the region, as not every ‘owner/guardian’ registers the AED they have purchased with WMAS as they are not legally required to do so. Although, there is no evidence to suggest it, this registration could be disproportionately lower in more deprived areas of the region. We have assumed that all AEDs were available for anyone to access at any time if an OHCA has occurred nearby.

## Data analysis

### Geocoding and mapping

Addresses of the OHCAs, schools and PAD sites were geocoded using the online resource Doogal (Batch geocoding (doogal.co.uk)) to produce the latitude and longitude for each location. Where this failed, Google Maps© was used to produce the latitude/longitude of the building or centre of the postcode.

The geocoded locations of OHCAs, schools and registered PADs was presented as maps using ArcGIS10.5 (ESRI, https://www.arcgis.com/index.html). The proximity analysis feature of ArcGIS calculated the number of OHCAs occurring within 100 m radii intervals of a school. The programme utilised the geodesic distances between features, accounting for the earth’s curvature.

### Statistical analysis

Descriptive statistics were used for the incidence and demographics of cardiac arrests and the school survey responses. Descriptive data were analysed using a Chi-squared test to compare proportions within the sample. A *p*-value of < 0.05 was considered statistically significant.

### Economic analysis

Economic analysis was based on a model developed by Anderson and his colleagues in Denmark, and full details of the model have been previously described^21^. Modelled health-states, utilities and the movement between health-states were unchanged but the population analysed was OHCAs occurring within a 300 m radius of a school in the West Midlands. A distance of 300 m was chosen because this was approximately the average AED retrieval radius used by all English Ambulance Trusts^22^. The analysis compared the cost-effectiveness of the use of school-based AEDs compared to no AED usage.

The base case analysis was from a societal perspective, accounting for lifetime costs and health outcomes measured in quality-adjusted life years (QALYs). Analyses were performed in TreeAge Pro 2018 Suite. Results are presented as costs per QALY gained. All costs were presented in 2019 Great British pounds (£).

Summaries of the data used to inform the model can be found Tables A1 and A2 in the appendix along with a summary of the economic costs. Model inputs were based on previously published literature for the annual hospitalisation costs associated with cardiac arrests23., 24., 25..

Annual hospitalisation costs associated with the initial cardiac arrest event were taken from a study by Petrie et al.^23^ and ranged from £35,295 per annum (for survivors to hospital discharge with a CPC score of 1 and 2) to £52,615 per annum (for survivors to hospital discharge with a CPC score of 3 and 4). Hospitalisation costs for those who did not survive to hospital discharge was £12,640. Annual post hospitalisation costs were estimated based on data from Paramedic-2 trial and ranged from £7,907 for patients with a good outcome to £24,457 for those with a poor outcome at hospital discharge.^25^

Maintenance and staff training were not included in the cost calculations as AEDs normally require minimal maintenance and are designed for use without formal training. For each OHCA occurring outside of a school we assumed that there was no overlapping AED coverage.

### Ethics

The OHCAO project has ethical approval from the National Research Ethics Service (ref: 13/SC/036) and the Confidential Advisory Group (CAG) (ref: ECC8-04(C)/2013). This project was granted ethics approval by the University of Warwick Biomedical & Scientific Research Ethics Committee (ref: REGO-2017-2107 AMO3).

## Results

### Public-access AED locations

The WMAS database contained 3,895 active AED locations, 5 of which contained incorrect or incomplete address information. In total 3,890 AEDs were mapped. According to the WMAS database 307 of 2,634 schools (11.7%) had an AED on-site.

### OHCA data

There were 11,411 OHCAs in the three-year study period. Twelve OHCAs were excluded as no location was provided on the registry and therefore could not be geolocated. Table 1 contains details of the 39 OHCAs occurring on or immediately outside school premises. School-based OHCAs accounted for 0.34% of all OHCAs in the 3-year study period, an incidence of 0.2 per 100,000 people in the general population per year. The rate of school-based OHCA was 4.9 per 1,000 schools per year.

**Table 1:** Demographics and outcomes of out-of-hospital cardiac arrests (OHCA) in schools in region served by West Midlands Ambulance Service University NHS Foundation Trust (n (%)) and region as a whole.

	School-based OHCAs (n = 39)	OHCAs in region (n = 11,399)
Age of OHCA:		
≤18 years	8 (20.5)	308 (2.7)
>18 years	31 (79.5)	11,103 (97.3)
Male	33 (84.6)	7209 (63.2)
Time of OHCA:		
Within school hours (08:00–17:30)	21 (53.8)	2152 (18.9)
Outside of school hours and weekends	18 (46.1)	9259 (81.1)
AED available^1^	8 (20.5)	555 (4.9)
AED used^1^	9 (23.0)	288 (2.5)
Survival status:		
Survived to hospital discharge	16 (41.0)	941 (8.2)
Survival unknown	7 (18.0)	871 (7.6)
Did not survive	16 (41.0)	9599 (84.1)

1 The reason why the AED used number is greater than AED available one is probably because an AED may have been used but was not registered with WMAS so they could not indicate it was available.

About 53.8% (n = 21) of school-based OHCAs occurred within school hours (presumed 08:00–17:30). The remaining 46.1% (n = 18) OHCAs occurred on weekdays outside of school hours (28.2%, n = 11) or at weekends (17.9%, n = 7).

An AED was known by WMAS to be present in 8 (20.5%) of school-based OHCAs, however, an AED was used in 9 (23.0%) OHCAs (regardless of AED presence being noted by EMS). Those aged < 18 years accounted for 20.5% of school-based OHCAs (p < 0.001), with 87.5% of these occurring within school hours; the one occurring outside of school hours was on a campus-based educational facility (p < 0.001). Sixteen (41.0%) patients survived to hospital discharge, whilst a similar proportion did not survive (p < 0.001).

### AEDS in schools survey

We obtained email addresses for 2,453 of 2,634 schools (93.0%). The remaining 184 email addresses were unavailable, and 36 emails were returned to sender. We contacted 2,417 of 2,634 schools (91.6%) successfully. A total of 323 schools replied (12.2% of all schools; 13.4% schools contacted, 2,417 contacted, 2,453 emailed), 184 (57.0%) indicated they had an AED on site, whereas 139 (43.0%) said they did not (p < 0.001) (Table 2).

**Table 2:** School automated external defibrillator (AED) survey responses.

Survey response	n (%)
Total emailed	2,453
Email successful	2,417 (98.5%)
Number of responses	323 (13.4%)
AED currently not present	139 (43.0)
AED present	184 (57.0)
Registered with WMAS	64 (34.8)
Not registered with WMAS	120 (65.2)

Of the 184 schools with an AED, 136 (73.9%) provided AED availability; 24 of the AEDs (13.0%) were confirmed to be available 24 h/day, 7-days/week. In our sample, the median school opening time was 08:00 and median closing time 17:30. On average, school-based AEDs were available for 12.4 h/day (including those available 24 h/day) and 9.9 h/day (excluding those available 24 h/day). Only 34.8% of AEDs were registered with WMAS.

### OHCAs within school locality

Table 3 gives the number of OHCAs that occurred within specified radii of a school. A total of 425 (3.7%) OHCAS occurred within 100 m, 4,250 (37.3%) within 300 m and 7,900 (69.3%) within 500 m.

**Table 3:** Number (%) of all-location out-of-hospital cardiac arrests (OHCA) occurring within a radius of a school in 100 m intervals that occurred between 2014 and 2016.

Radius distance to school^1^	Cumulative number of OHCAs (n, %)	Number of schools within radius of an OHCA (N, %)
In school	39 (0.3)	-
100 m	425 (3.7)	354 (13.4)
200 m	2093 (18.4)	1320 (50.1)
300 m	4250 (37.3)	1927 (73.1)
400 m	6365 (55.8)	2215 (84.0)
500 m	7900 (69.3)	2319 (87.9)
600 m	9029 (79.2)	2387 (90.5)
700 m	9658 (84.7)	2415 (91.6)
800 m	10,020 (87.9)	2434 (92.3)
900 m	10,294 (90.3)	2453 (93.0)
1,000 m	10,459 (91.8)	2472 (93.7)
All	11,399	2637

1 Straight line distance.

### Economic analysis

Economic modelling calculated that a school-based AED programme would generate additional costs of £2,286 and additional QALYs of 0.26 over the lifetime of cardiac arrest survivors compared with no AED programme (Table 4). The incremental cost-effectiveness ratio (ICER) was £8,916 per QALY gained. The probability that a school-based AED programme is cost-effective at a £20,000 willingness-to-pay threshold is 0.92. In tested scenarios, the cost-effectiveness results were robust to changes in the annual incidence of OHCA per AED but sensitive to a change in bystander training costs.

**Table 4:** Cost-effectiveness analysis results for use of an automated external defibrillators on out-of-hospital cardiac arrests that occurred within 300 m of a school.

	IncrementalCosts (£)	Incremental QALYs	ICER (£/QALY)
Andersen base-case	9,852*	0.26	38,426*
UK school AED base-case	2,286	0.26	8,916
Bystander training costs changed from £0 to £15 per individual	11,929	0.26	46,527
Bystander training costs changed from £0 to £10 per individual	8,715	0.26	33,990
Annual incidence of OHCA per AED changed from 20% to 35%	2,092	0.26	8,160
Annual incidence of OHCA per AED changed from 35% to 10%	2,739	0.26	10,681
Hospital costs doubled	2,810	0.26	10,961
After hospital discharge costs doubled	3,595	0.26	14,024
Doubled the cost of AED used	2,739	0.26	10,681

* Assuming an exchange rate of £1 to $1.4.

## Discussion

In this study, 39 OHCAs occurred in schools, 31 (79.5%) of these in those over 18 years of age. Of the school-based OHCAs, 21 (53.8%) occurred within school hours and 16 (41.0%) survived to hospital discharge. In a survey of schools, 184 (57.0%) reported having an AED and 64 (34.8%) of these were registered with WMAS. Of all OHCAs occurring in the study period, 4,250 (37.3%) were within a 300 m radius of a school. Economic analysis calculated the incremental cost-effectiveness ratio (ICER) was £8,916 per QALY gained.

The observed incidence of 4.9 OHCAs per 1,000 schools per year compares with an incidence of 5.72 seen in South Korea^26^, 3.5 in Japan^27^, and 6.0 in Seattle and King County.^28^ But it is significantly lower than incidences of about 16^29^ and 21^30^ observed in Tennessee and about 14^14^ and 20^31^ in US high schools. This reinforces the observation that cardiac arrests rarely occur in schools.

In our study, school-based OHCAs were more likely to occur in adults than in children, with 79.5% of OHCAs occurring in those > 18 years of age. This compares internationally with studies reporting occurrences of between 55% and 73% of OHCAs in schools occurring in adults.^9^ This suggests that placing AEDs in schools affords benefit to both children attending the school and adult employees and visitors.

Survival to hospital discharge in this study was 41.0% in school-based OHCA, compared to 8.2% in the whole study population. This increased survival is likely attributable to factors such as OHCAs being more often witnessed by a bystander in a school setting, who promptly give CPR, and is comparable with other studies looking at school-based OHCAs.14., 27., 32., 33. In addition, it may be because cases were well enough to be out of their homes and at school at the time of their arrest.

Only 53.8% of school-based OHCAs in our sample occurred during school hours, when school-based AEDs are generally available. The survey highlighted that only 7% of school-based AEDs were available 24/7, leaving a substantial proportion of cardiac arrest victims without access to an AED, despite one being on site. Research on school-based AEDs in Copenhagen in 2013 reported that only 9.1% of all AEDs were available at all hours, with coverage decreasing by up to 53.4% during the evening, night-time and weekends.^34^.However, a more recent study noted a significant improvement in availability to 33.3%.^8^ This loss of accessibility can drastically decrease an AED’s utility considering the proportion of OHCAs occurring outside of school hours.

Of the schools who replied to the survey, 57.0% had an AED on site, only 34.8% of which were registered with the EMS. Whilst our school survey may not give a full picture of school-based AEDs across the West Midlands, the difference between the proportion of schools with an AED according to the WMAS PAD database (11.6%) and our survey (57%), coupled with only 34.8% of the survey sample reporting they had registered their AED, indicates a significant proportion of unregistered school-based AEDs. To improve AED registration in UK the British Heart Foundation and Microsoft announced a partnership to build a national cloud-based registry of all AEDs in the UK and to make this data available to all ambulance services (https://www.bhf.org.uk/what-we-do/news-from-the-bhf/news-archive/2018/August/the-bhf-joins-forces-with-microsoft-and-the-nhs-to-save-thousands-more-lives-from-cardiac-arrests).

We calculated that 37.3% of all OHCAs in the West Midlands region occurred within a 300 m radius of a school. Better registration rates with EMS and increasing accessibility and availability of school-based AEDs to the local community could make a significant contribution towards increasing AED coverage, use and survival rates from OHCAs. The Resuscitation Council UK has issued guidelines as to how to improve the accessibility and visibility of AEDs such as by using clear, nationally-agreed signage and not locking or placing them in inaccessible areas.^6^

Placing an AED in every school and restricting access has been shown not to be cost-effective.^35^ However, we have shown that the incremental cost-effectiveness ratio (ICER) of using school-based AEDs to treat community-based OHCAs was £8,916 per QALY gained, well below the £38,426 ($53,797) found by Anderson et al.,^21^ which may add weight to the argument for increasing the number of school-based AEDs available to the wider community 24 hours a day.^35^ Work in British Columbia, Canada added for this argument by observing that if AEDs were placed outside each school the placement surpassed the cost-effective threshold, even if there was incomplete utilisation.^36^

Our geographical analysis relied upon the accuracy of location data provided in the OHCAO registry, WMAS AED database and Department for Education schools database. The geographical software utilised straight-line distances between locations which, whilst accounting for the curvature of the earth, would not account for the distance travelled if utilising footpaths or roads. Calculating walking distance rather than straight line distance to the nearest AED will lead to a reduction in AED coverage for OHCAs.22., 37. Further geographical analysis will be more accurate if it utilises walking route distances. Our analysis utilised the mean opening and closing hours reported by schools, but future work could take into account term-time compared to holiday-time AED availability in schools.

## Limitations

This study has a number of limitations. We only looked at OHCAs that received treatment from EMS staff, the number of 999 calls received to attend a potential OHCA by dispatchers in the region is almost three-times the number of cases we analysed.^3^ The Utstein location was missing or not recorded in a large number of cases, however, we also used the OHCA location, where the full address of the OHCA event was given, to check for where a school was mentioned. This was also used to eliminate Universities from the analysis. As mentioned in the methods the WMAS AED database is not complete because it is well known that a significant number of AEDs are not registered. However, if the number registered increased it would probably result in an increase in the number of OHCAs covered. The school survey response rate was very poor probably because the email sent out was unsolicited. In addition, schools with an AED may have been more likely to have responded to the survey, especially those who have registered the AED with WMAS. In the cost-effective analysis we applied the probabilities and costs used by Andersen et al.^38^ These may not be strictly applicable to the English situation, where the health system is different from that in the USA. However, to some extent we did make some adjustments in costs (see Appendix Tables A1 and A2).

## Conclusion

Our study confirms previous findings that OHCAs in school are rare and more likely to occur in adults than in children. As 37.3% of all-location arrests occurred within 300 m of a school, and with English PAD usage rates at 2.3%^2^ there is great potential for increasing the use of AEDs. Accessibility to school-based AEDs could be improved by increasing accessibility and improving registration with Emergency Medical Services. A strategy of placing AEDs in schools is likely to be cost-effective.

## CRediT authorship contribution statement

**Madeleine Benson:** Conceptualization, Methodology, Formal analysis, Writing – original draft, Visualization, Project administration. **Terry P. Brown:** Conceptualization, Methodology, Software, Validation, Writing – original draft, Writing – review & editing, Visualization. **Scott Booth:** Conceptualization, Methodology, Software, Validation, Data curation, Writing – original draft, Writing – review & editing, Visualization. **Felix Achana:** Methodology, Software, Validation, Formal analysis, Writing – original draft, Writing – review & editing, Visualization. **Christopher M. Smith:** Software, Validation, Writing – review & editing. **Gill Price:** Data curation, Writing – review & editing. **Matt Ward:** Data curation, Writing – review & editing. **Claire Hawkes:** Validation, Writing – review & editing. **Gavin D. Perkins:** Conceptualization, Writing – review & editing.
