# Supplementary material for: Location of out-of-hospital cardiac arrests and automated external defibrillators in relation to schools in an English ambulance service region
Source: Resusc Plus. 2022 Jul 26;11:100279. doi: 10.1016/j.resplu.2022.100279 (PMC9335389; doi:10.1016/j.resplu.2022.100279)
Supplement: Supplementary data 1 [file mmc1.docx]

# Appendix

| Table A1: Data (Probabilities) used to inform economic modelling as compared to that used in Anderson et al. (13). | | |
| --- | --- | --- |
| Parameter | Base case  estimate^a^ | Base case  (This study) |
| Incidence of OHCA per school AED per year (%) | 20 | 35 |
| AED used when present (%):   - Bystander trained (%) - Bystander not trained | 50  10 | 50  10 |
| Proportion shockable (%):   - No AED group - AED group | 48  53 | 48  53 |
| Short-term outcomes (%):   - Favourable functional outcome in the no AED group:   - Shockable   - Non-shockable - Risk ratio for favourable functional outcome in the AED group:   - Shockable   - Non-shockable | 34  7  1.52  1 |  |
| CPC score at hospital discharge | eTable 10^a^ | eTable 10 ^a^ |
| Long-term outcomes: (%)   - Post-discharge yearly mortality stratified by CPC — the first year: - Post-discharge yearly mortality stratified by CPC — subsequent years: | 1: 9  2: 23  3: 36  4: 72  1: 5  2: 8  3: 9  4: 5 | 1: 9  2: 23  3: 36  4: 72  1: 5  2: 8  3: 9  4: 5 |
| Utilities stratified by CPC: | 1: 0.77  2: 0.49  3: 0.32  4: 0 | 1: 0.77  1: 0.49  1:0.32  1: 0 |

| Table A2: Data (Costs per cardiac arrest) used to inform economic modelling as compared to that used in Anderson et al. (13). | | | |
| --- | --- | --- | --- |
| Parameter | Base case estimate(13) | Base case (This study) | Source/comment |
| Yearly cost of an AED | $255 | £181 |  |
| Bystander AED training | $19,286 | £0 |  |
| Emergency department costs | $2,643 | 0 (included in hospital costs) |  |
| Hospital costs stratified by discharge CPC | 1: $89,027  2: $100,469  3+4: $130,846  5: $37,262 | 1: £37,499  2: £37,499  3+4: £55,900  5: £13,429(15) | (95% CI £9,056.87-141,915.74)  (95% CI £9,056.87-141,915.74)  (95% CI £40,371.29-56,454)  (95% CI £1,997.43-42,233.97) |
| Costs after hospital discharge stratified by discharge CPC — first year | 1: $24,857  2: $46,276  3: $83,356  4: $13,918 | 1: £19,202  2: £19,202  3: £27,882  4: £27,882(16) |  |
| Costs after hospital discharge — subsequent years | $5,761 | £3,187 | Assumed to be same as first-year costs stratified by functional status |

## Costs of school-based AED programme

The cost of a school-based AED programme was estimated using the methodology described by Anderson et al (13) and included (i) the acquisition cost of the device, storage cabinet (assuming that devices are available 24 hours a day) and initial installation, and (ii) ongoing maintenance and or usage costs.

In the UK, the Department for Education in collaboration with the Department of Health has put in place arrangements to enable schools to purchase AEDs through the NHS Supply Chain at a reduced cost (10). A device purchased through such arrangements would cost £617.57 in 2017 prices inclusive of VAT and £701.78 in 2018 taking into account hospital and community health services price inflation (28). The cost of a wall mounted cabinet with alarm is £126 inclusive of VAT 2019 prices (the defib) for the Mediana HeartOn A15 defibrillator, the model cited in the Department for Education guidance (<https://www.thedefibpad.co.uk/mediana-hearton-a15-defibrillator-alarmed-wall-cabinet/>). Initial installation of the device is estimated to cost £17 based on the assumption that the work is carried out by a Band 5 emergency paramedic/ambulance staff costing £34 an hour (28) and take no more than 30 minutes to complete (13). Next is the cost of ongoing usage and maintenance costs which we assumed to involve replacing the electrode pads and batteries whenever the device is used or every 4 years (based on a device shelf-life of 3-5 years) (4). It was assumed that the replacement of pads and batteries would be undertaken by a competent paramedic, taking no more than 15 minutes of their time at a cost of £8.50 per activity. Since AEDs are meant to be used in cases of cardiac arrest, we model the cost of replacing pads and batteries following usage as a function of number of cardiac arrest events occurring within 300 metres of a school with the assumption that every AED is used for every event.
